# Defective lysosomal proteolysis and axonal transport are early pathogenic events that worsen with age leading to increased APP metabolism and synaptic Abeta in transgenic APP/PS1 hippocampus

**DOI:** 10.1186/1750-1326-7-59

**Published:** 2012-11-22

**Authors:** Manuel Torres, Sebastian Jimenez, Raquel Sanchez-Varo, Victoria Navarro, Laura Trujillo-Estrada, Elisabeth Sanchez-Mejias, Irene Carmona, Jose Carlos Davila, Marisa Vizuete, Antonia Gutierrez, Javier Vitorica

**Affiliations:** 1 Instituto de Biomedicina de Sevilla (IBIS), Hospital Universitario Virgen del Rocio, Consejo Superior de Investigaciones Cientificas Universidad de Sevilla, c/ Manuel Siurot s/n, 41013, Sevilla, Spain; 2 Department Bioquimica y Biologia Molecular, Facultad de Farmacia, Universidad de Sevilla, Sevilla, 41012, Spain; 3 Centro de Investigacion Biomedica en Red sobre Enfermedades Neurodegenerativas (CIBERNED), Madrid, Spain; 4 Department Biologia Celular, Genetica y Fisiologia, Facultad de Ciencias, Universidad de Malaga, Malaga, 29071, Spain

**Keywords:** Alzheimer’s disease, PS1/APP transgenic model, Dystrophic neurites, Tau phosphorylation. Cathepsin activity, APP processing, Abeta production

## Abstract

**Background:**

Axonal pathology might constitute one of the earliest manifestations of Alzheimer disease. Axonal dystrophies were observed in Alzheimer’s patients and transgenic models at early ages. These axonal dystrophies could reflect the disruption of axonal transport and the accumulation of multiple vesicles at local points. It has been also proposed that dystrophies might interfere with normal intracellular proteolysis. In this work, we have investigated the progression of the hippocampal pathology and the possible implication in Abeta production in young (6 months) and aged (18 months) PS1(M146L)/APP(751sl) transgenic mice.

**Results:**

Our data demonstrated the existence of a progressive, age-dependent, formation of axonal dystrophies, mainly located in contact with congophilic Abeta deposition, which exhibited tau and neurofilament hyperphosphorylation. This progressive pathology was paralleled with decreased expression of the motor proteins kinesin and dynein. Furthermore, we also observed an early decrease in the activity of cathepsins B and D, progressing to a deep inhibition of these lysosomal proteases at late ages. This lysosomal impairment could be responsible for the accumulation of LC3-II and ubiquitinated proteins within axonal dystrophies. We have also investigated the repercussion of these deficiencies on the APP metabolism. Our data demonstrated the existence of an increase in the amyloidogenic pathway, which was reflected by the accumulation of hAPPfl, C99 fragment, intracellular Abeta in parallel with an increase in BACE and gamma-secretase activities. In vitro experiments, using APPswe transfected N2a cells, demonstrated that any imbalance on the proteolytic systems reproduced the in vivo alterations in APP metabolism. Finally, our data also demonstrated that Abeta peptides were preferentially accumulated in isolated synaptosomes.

**Conclusion:**

A progressive age-dependent cytoskeletal pathology along with a reduction of lysosomal and, in minor extent, proteasomal activity could be directly implicated in the progressive accumulation of APP derived fragments (and Abeta peptides) in parallel with the increase of BACE-1 and gamma-secretase activities. This retard in the APP metabolism seemed to be directly implicated in the synaptic Abeta accumulation and, in consequence, in the pathology progression between synaptically connected regions.

## Background

Alzheimer´s disease (AD) is a proteinopathy characterized by the accumulation of aggregated extracellular amyloid-beta (Abeta, Aβ) peptides and intracellular hyperphosphorylated tau (revised in [1]). Concomitant with appearance of extracellular Abeta deposits, another central pathological feature of the disease is the early formation of amyloid plaque-associated neuritic changes in the form of dystrophic neurites, together with a selective loss of connections and neuronal groups. Dystrophic neurites, defined as thickened or irregular neuronal processes, are considered to be expression of a widespread alteration of the neuronal cytoskeleton. In AD, dystrophic axons are particularly abundant in the hippocampal fiber systems originating from the subiculum, CA1, and the entorhinal cortex [2]. However the exact molecular mechanisms underlying the pathogenesis of AD remain to be elucidated.

Dystrophic neurites were characterized by the presence of numerous vesicles of multiple origins [3,4]. Several lines of investigation support the notion that defective autophagy process, a cellular catabolic mechanism essential for degradation of aggregated proteins and organelles, significantly contributes to AD pathogenesis [5-8]. Interestingly, autophagic compartments have been reported to participate in APP processing and Aβ peptides production [9].

Abeta peptides, cytotoxic in their oligomeric state [10-13] derive from the sequential cleavage of APP by beta- and gamma-secretases [14,15]. Although the exact intracellular localization of APP processing is unknown, the autophagic and endocytic pathways could be both involved in precursor protein (APP) processing and Abeta generation. In this sense, BACE-1 and gamma-secretase complex have been detected in many cellular locations, including early and late endosomes [16], autophagic vacuoles [17-19] and lysosomes [20]. On the other hand, the Abeta degradation, in vivo, could be mediated by several proteases, as neprilysin, IDE, and several cathepsins as B, D and E [21]. Abnormal processing of APP or Abeta accumulation in AD could be related to several mechanisms, including excessive production, abnormalities in transport, alteration of autophagic and endosomal pathways, and deficits in its degradation through the lysosome or the ubiquitin-proteasome system (UPS) [22-24]. In fact, accumulation of autophagic vacuoles (AVs) has been observed in brains from AD patients [3,19] and in PS1/APP mice after Abeta deposition [17,19,25]. Moreover, the AVs were principally accumulated within dystrophies and could reflect impairment in AVs clearance in AD brains [5]. In this sense it has been reported that enhancing lysosomal cathepsin activity ameliorates Abeta toxicity [26] and restoring the autophagy-lysosomal pathway (by deletion of cystatin B) reduced amyloid load and rescued memory performance [9].

In the present work we investigated how the possible age-related relationship between aberrant Abeta generation and dysfunctions in axonal cytoskeleton as well as in lysosomal and proteasomal systems, manifested in our PS1/APP AD model. We proposed that the decrease in lysosomal proteolytic activities was implicated in increased Abeta production. Abnormal accumulation of Abeta could aggravate the axonal and cytoskeleton abnormalities linked to the pathology of AD.

## Results

### The age-dependent increase of neuritic dystrophies was paralleled by phosphorylation of cytoskeletal proteins and decrease in motor proteins

**Figure 1:**
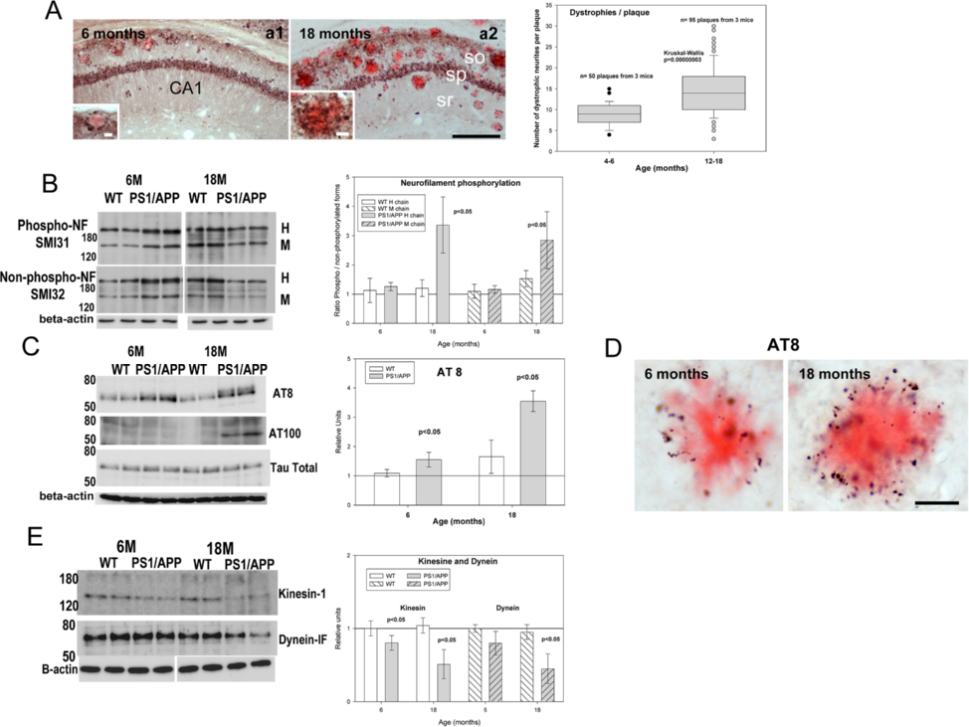
**Aged-dependent increase of the hippocampal axonal pathology in PS1/APP mice.A**) APP-immunolabeled CA1 sections with Congo red at 6 (left) and 18 (right) months of age showing the age-dependent increase in the dystrophic neurite pathology. Dystrophies were concentrated surrounding Abeta plaques (insets in a1 and a2 show higher magnifications). The quantitative analysis of plaque-associated dystrophies demonstrated a significant increase in aged mice. **B**) Representative western blots (n=5/age/group) for phosphorylated (upper panel) and non-phosphorylated (lower panel) neurofilament (SMI antibodies). Bands corresponding to high (H) and medium (M) chains were indicated. Quantitative analysis revealed an increase in the phosphorylated vs non-phosphorylated H and M neurofilaments in aged PS1/APP mice. No changes were observed in 6 month-old PS1/APP or WT mice. **C**) Tau phosphorylation was determined by western blots using AT8 (upper panel), AT100 (medium panel) and tau (lower panel) antibodies at 6 and 18 months of age (n=5/age/group). Graph showed the quantitative analysis of AT8 western blots. Due to the lack of immunoreactivity in WT and 6 months PS1/APP mice, the AT100 signal could not be quantified. A prominent increase in AT100 epitope (absent in other conditions) was observed in 18 month-old transgenic mice. **D**) AT8 immunostaining revealed that phospho-tau concentrated principally in dystrophies around congophilic plaques. **E**) Western blots and quantitative analysis (n=5/age/group) of kinesin (upper panel) and dynein (lower panel) expression. Both motor proteins decreased in PS1/APP mice. So, stratum oriens; sp, stratum pyramidale; sr, stratum radiatum. Scale bars: 200 μm (a1/a2), 10 μm (inset a1), 20 μm (inset a2), 20 μm (D).

APP-positive dystrophic neurites represented an early pathology in our PS1/APP model [17]. These dystrophies were of axonal origin and, at early ages, were located exclusively surrounding congophilic Abeta plaques. It was also known that Abeta load increased with aging in this and most AD models [27]. Thus, we have first evaluated the progression of the APP-positive dystrophy formation from early (4–6 months) to late (12–18 months) ages. As expected, the number of APP-positive dystrophies, surrounding Abeta plaques, increased significantly in 12–18 month cohort (Figure 1A), paralleling the increase in plaque size and number (Figure 1A and [17]). Furthermore, according with previous data, the dystrophic neurites were predominantly concentrated surrounding the congophilic Abeta plaques at all ages.

The presence of aberrant hyperphosphorylated cytoskeletal proteins is one of the major pathological hallmarks of AD [28]. Furthermore, hyperphosphorylated neurofilaments and tau seemed to overlap with senile plaques in AD patients and models (unpublished data, see also [17,28,29]). Thus, hyperphosphorylated cytoskeletal proteins could also be implicated in the progression of the pathology in our PS1/APP model. To determine this possibility, we tested the neurofilaments (using SMI antibodies) and tau phosphorylation (AT8 and AT100 epitopes) in 6- and 18 months PS1/APP. As we described previously [17], 6-month-old PS1/APP mice displayed only minor modifications. In fact, as compared with age-matched WT mice, neurofilament (heavy and medium chains; H, M) phosphorylation (calculated as ratio between phospho and non-phosphorylated neurofilament) exhibited no apparent modifications at this age (see Figure 1B). Only the levels of AT8 epitope were slightly but significantly increased. However, 18-month-old PS1/APP mice displayed advance cytoskeletal pathology. As shown (Figure 1B), neurofilament heavy and medium chains were hyperphosphorylated (mostly due to a decrease in the non-phosphorylated forms). In agreement with this observation, both AT8 and, more prominently, AT100 tau phosphoepitopes (Figure 1C) were increased in 18 month-old PS1/APP. The presence of hyperphosphorylated tau was further confirmed by immunohistochemistry. Tau-reactive (AT8) dystrophic neurites, localized surrounding amyloid plaques, were detected since early ages (Figure 1D).

To further evaluate whether microtubule vesicular transport might be compromised in PS1/APP mice, we have assessed the levels of kinesin-1 and dynein motor proteins. As previously reported [17], 6 month-old PS1/APP mice showed a modest reduction on both proteins and, in accordance with the advance pathology, the levels of both motor proteins were dramatically reduced in 18 months PS1/APP mice (Figure 1E).

These data confirmed and extended our previous observation in this model, at early ages, and demonstrated the existence of a clear age-dependent axonal pathology implicating cytoskeletal and motor proteins.

### The age-dependent progression of the neuritic pathology was associated to an impairment of proteolysis mechanisms

**Figure 2:**
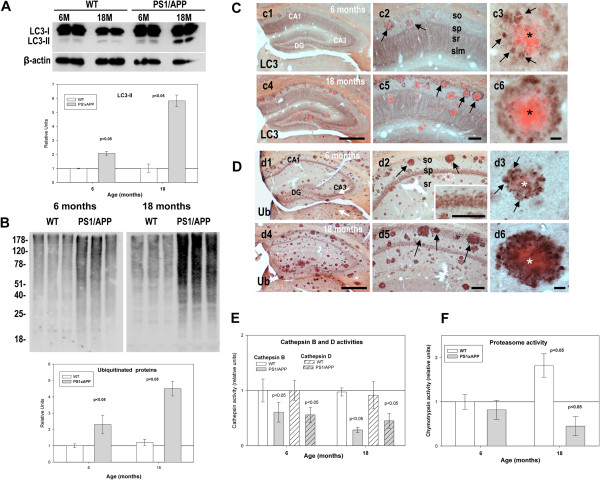
**Impairment of lysosomal and proteasomal protein-degradation in PS1/APP mice.** A and B; Representative western blots and quantitative analysis (n=5 for each age and animal group) of the autophagic vesicle marker LC3 (**A**) and ubiquitinated proteins (**B**) performed using the same protein samples. An age-dependent accumulation of both LC3-II and ubiquitinated proteins were observed exclusively in PS1/APP samples. (**C-D**) Three different magnifications of LC3- (**C**) or ubiquitin- (**D**) immunolabeled hippocampal sections (counterstained with Congo red for Abeta plaques) of 6 and 18 months of age PS1/APP mice corroborated the age-dependent accumulation of both markers, associated with dystrophic neurites surrounding amyloid plaques (c1, c4, d1 and d4, panoramic views of immunostained hippocampus; c2, c5, d2 and d5, CA1 region, arrows indicate plaques covered by immunopositive dystrophies and inset in d2 shows immunolabeling in the pyramidal layer somata; c3, c6, d3 and d6, representative image of a congophilic plaque surrounded by dystrophic neurites, arrows point to positive dystrophies around a congophilic plaque indicated by an asterisk). Assays of lysosomal cathepsin B or D (**E**) and proteasomal chymotrypsin-like (**F**) enzymatic activities demonstrated the existence of a pronounced decrease in cathepsin B and D activities (since early ages in PS1/APP), whereas proteasome was affected only in aged tg mice. CA1, hippocampal subfield; DG, dentate gyrus; so, stratum oriens; sp, stratum pyramidale; sr, stratum radiatum; slm, stratum lacunoso-moleculare. Scale bars: 500 μm (c1/c4, d1/d4), 100 μm (c2/c5, d2/d5), 10 μm (c3/c5, d3/d5); 50 μm (d2, inset).

It has been reported that axonal dystrophy could be produced by inhibition of the lysosomal proteolysis [30,31] or by axonal transport deficiencies [32]. However, axonal transport was also essential for the correct lysosomal maturation and intracellular protein degradation [33]. Thus, the formation and the age-dependent increase in neuritic dystrophies could be cause or consequence of a progressive reduction of the intracellular proteolytic processes. Theoretically, an impairment on either proteasomal and/or autophagic/lysosomal route, in the PS1/APP model, should be reflected by the accumulation of ubiquitinated proteins and/or the autophagic maker LC3-II. In fact, our data (Figures 2A and B) clearly demonstrated the existence of a marked and early accumulation of both ubiquitinated proteins and LC3-II in hippocampal samples from PS1/APP mice. This accumulation was observed since early ages (6 months of age), increased significantly in aged PS1/APP mice (18 months) and was not observed at 2 months of age, before plaque deposition (not shown). On the contrary, WT mice displayed absolutely no changes at these ages. These data were further confirmed by immunohistochemistry experiments. As shown, (Figure 2C, c1 to c6), the LC3 immunoreactivity was principally located in the somata and apical dendrites of principal neurons whereas ubiquitin immunostaining (Figure 2D, d1 to d6) was mainly located at the cell bodies (see Figure 2D, d2 inset). Furthermore, most (if not all) Abeta plaques (stained with Congo Red) were surrounded by dystrophies, LC3 or ubiquitin immunopositive, both at 6 months and more patently at 18 months of age.

Although the accumulation of LC3-II could reflect both, induction in the autophagy route or decrease in the autophagosome degradation, the presence of ubiquitinated proteins in a similar localization of LC3, surrounding Abeta plaques, strongly suggested the existence of a decrease in autophagosome degradation. Among different causes, the decrease in autophagosome degradation could reflect a decrease in the lysosomal activity. Thus, we next evaluated the lysosomal and proteasomal activity by determining the cathepsins, B and D, activities and the proteasomal chymotrypsin-like activity. As shown (Figure 2E), both cathepsin B and D activities decreased since relatively early ages. Young (6 months) PS1/APP displayed a consistent (−39.21±18.84%; n= 5, p<0.05; -44.15±13.51%, n=3, p<0.05; for cathepsin B and D, respectively) decrease (as compared with age matched WT) whereas 18 month-old PS1/APP mice displayed a further diminution (−72.50±4.2%; n=4, p<0.05; -55.45±6.7, n=3, p<0.05, respectively). No changes were observed before plaque deposition (at 2 month-old, not shown). On the other hand, the proteasomal activity (Figure 2F) also displayed a slight, and not significant diminution at 6-months (−20.4±12.3%, n=4) followed by a more pronounced inhibition in aged PS1/APP mice (−45.5±15.6%; n=4; p<0.05). No differences between WT and PS1/APP were observed before the plaque deposition (2 months of age, not shown) and no inhibition was observed in 18 months WT mice.

**Figure 3:**
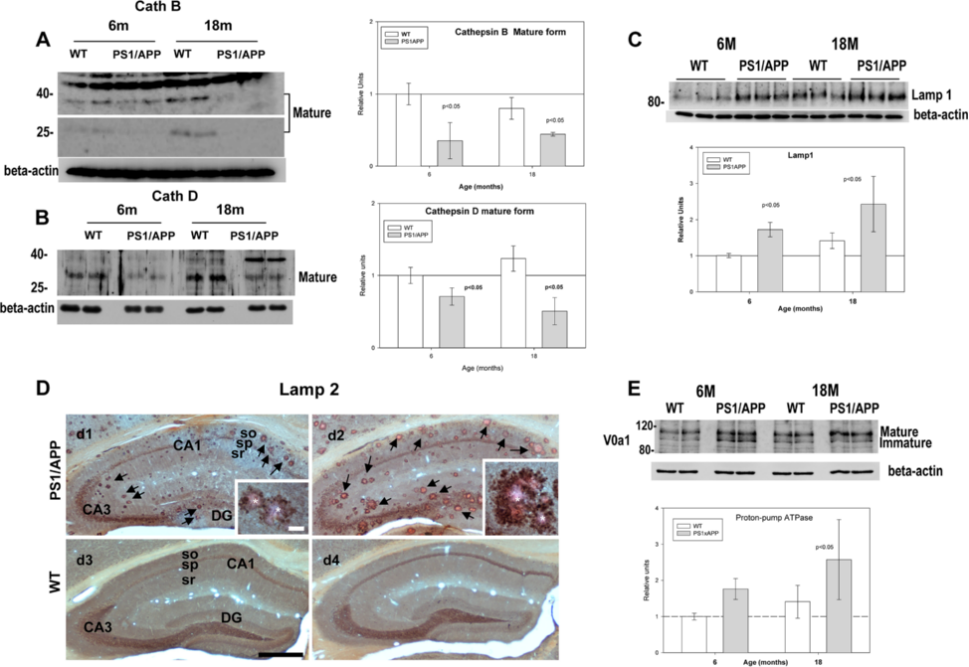
**The mature forms of cathepsin B and D decreased whereas Lamp1, Lamp2 and V0a1 proton pump subunit accumulated in PS1/APP mice hippocampus.** (**A**-**B**) Western blots and quantitative analysis (n=5 for each age and animal group) of cathepsin B (**A**) and D (**B**) showed the existence of a significant decrease in the mature forms of both proteins. This decrease was observed exclusively in PS1/APP transgenic mice at both ages tested (6 and 18 months). **C**-**D**) Same mice model accumulated Lamp1 (tested by western blot, C, n=3) or Lamp2 (tested by immunohistochemistry at 6 and 18 months of age). Lamp2 immunolabeled sections of 6 (d1) and 18 (d2) month-old PS1/APP mice counterstained with Congo red for fibrillar amyloid plaques revealed Lamp2 labeling associated to dystrophic neurites surrounding amyloid plaques (arrows) at both ages that increased with age. In WT mice (d3 and d4, for 6 and 18 months respectively) Lamp-2 immunostaining was associated to cell somata and neuropile. No dystrophies were detected in control samples. **E**) Representative western blot and quantitative analysis (n=5 for each age and animal group) of V0a1 proton pump subunit in samples from 6 and 18 month-old WT and PS1/APP mice. Both, the mature and immature forms of V0a1 seemed to be accumulated, although not significantly, in 6 and 18 months of age PS1/APP mice. CA1, hippocampal subfield; DG, dentate gyrus; so, stratum oriens; sp, stratum pyramidale; sr, stratum radiatum. Scale bars: 500 μm (**D**), 25 μm (insets in d1 and d2).

Consistent with the decrease in lysosomal activity, we also observed a significant reduction in the mature forms of both cathepsins B and D in PS1/APP mice (Figures 3A and B) and an accumulation of the lysosomal protein lamp 1 (Figures 3)C,at 6 and 18 months. Furthermore, this accumulation of lysosomal proteins seemed to be localized in dystrophies surrounding Abeta plaques, as demonstrated by immunohistochemistry analysis of lamp 2 protein (Figure 3D). These data revealed the existence of a lysosomal dysfunction in PS1/APP mice after plaque deposition. In this sense, it has been recently reported [6] that PS1 mutations affected the correct maturation of the V0a1 subunit of the vATP-ase and the lysosomal acidification. However, in our model, we observed an accumulation of the mature form of V0a1 protein (Figure 3E), similar to lamp 1 protein (compare Figures 3C and E). Furthermore, 6-month-old homozygous PS1M146L transgenic mice displayed no alterations on cathepsin B activity (not shown).

### The amyloidogenic APP processing and both BACE-1 and gamma-secretase activities were increased in aged PS1/APP mice

**Figure 4:**
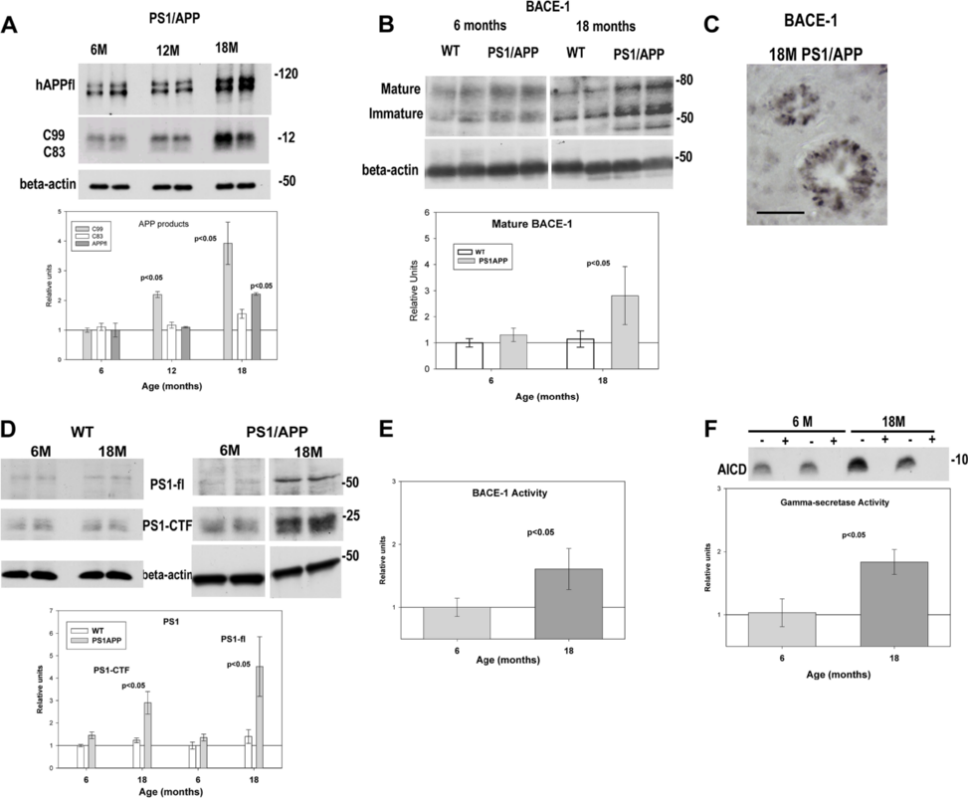
**Increased amyloidogenic processing of APP in aged PS1/APP hippocampus.A**) Representative western blots (n=4 for each age and animal group) for full-length APP (hAPPfl) (upper panel) and, APP-C terminal fragments (C99 and C83; middle panel) in 6, 12 and 18 month-old PS1/APP hippocampal samples. Quantitative analysis showed a significant increase in C99 from 6 to 18 months. APPfl also displayed a moderate (but significant) accumulation at 18 month-old. The amyloidogenic route was evaluated by testing the expression of BACE-1 (by western (**B**), n=5; and immunohistochermistry (**C**), n=3) and PS1 (n=6) (**D**) proteins. Dystrophic neurites surrounding plaques were positive for BACE-1 antibody. The enzymatic activities of BACE-1 (n=3) (**E**) and gamma-secretase (n=6) (**F**) were also assayed. Inset in panel F showed the accumulation of soluble AICD after 2 hours of incubation (at 37°C), in absence (minus) and in presence (plus) of 100 μM of the gamma-secretase inhibitor L-685-458, demonstrating that AICD production was dependent of the gamma-secretase activity. As shown, 18 month-old PS1/APP mice displayed an accumulation of both BACE-1 and PS1 (CTF fragment and full length) proteins paralleled by an increase in the corresponding enzymatic activities. Scale bar: 50 μm.

Data so far demonstrated the existence of a progressive age-dependent neuritic (probably axonal) pathology in the PS1/APP model that affected the intracellular proteolytic systems. We next evaluated whether this progressive pathology could affect the amyloidogenic APP processing. As shown (Figure 4A), the mature hAPPfl (visualized as two bands, corresponding with the mature and immature forms of the transgenic hAPP) significantly increased in the hippocampus of 18 month tg mice, as compared with 6 month-old animals (2.01±0.04; n=6; p<0.05), Also, a significant increase in the ratio mature/immature form of the hAPP was observed (data not shown). However, no differences in mRNA expression of the transgenic hAPP were detected between 2 and 18 months (data were normalized to the expression levels observed in 6 months PS1/APP, n=6 for each age, 1.12±0.15, 1.0±0.27, 1.33±0.29 and 1.23±0.48 for 2, 6, 12 and 18 month PS1/APP, respectively).

**Figure 5:**
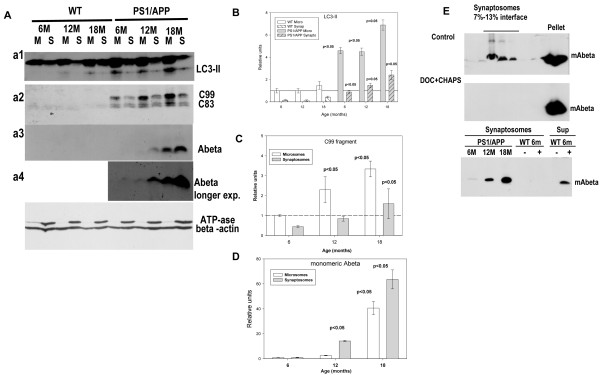
**In vitro inhibition of either lysosomal and/or proteasomal activities induced the amyloidogenic APP processing in N2a-APPswe cultures.A**) The inhibition of proteasomal/lysosomal (MG132) or lysosomal (Chloroquine) activities produced the accumulation of both ubiquitinated proteins (upper panel) and LC3-II autophagosomal marker (middle panel). Graphs showed the quantitative data of three different experiments using three different N2aAPPswe cultures. **B**) Using the same protein samples as in (**A**), we analyzed the effect of either inhibitor on the APP-CTF processing (upper western) and intracellular Abeta accumulation (lower western). As also shown quantitatively in graphs, either treatment produced the increase in the APP processing and the intracellular Abeta accumulation. **C**) In parallel, the expression of BACE-1 and PS1-ctf was also tested. As showed quantitatively, after 24h of treatment, both BACE-1 and PS1-cft were significantly accumulated in N2aAPPswe cells.

The APP proteolytic fragments C83 (alpha-CTF) and C99 (beta-CTF) are products from alpha- or beta-secretase processing, respectively, and substrates for gamma-secretase complex. As also shown in Figure 4A, the levels of C99 showed a clear age-dependent increase, from 6 to 18 month in PS1/APP mice whereas C83 levels were low (respect to C99) and remained practically unaltered during aging (see also Figure 5). Together with the accumulation of APP and APP-fragments, we also observed an increase in the levels of both BACE-1 and PS1 proteins. As shown, Figure 4B, the mature (and immature) form of BACE-1 was increased in 18-month-old PS1/APP mice (as compared with 6 months) whereas no differences in mRNA levels were observed (data were normalized by the expression of 6-month-old WT mice, n=5 for each age and genotype: 6 months: 1.00±0.08 vs 0.88±0.24; 12 months: 1.39±.19 vs 1.10±0.12; 18 months: 1.29±0.20 vs 1.33±0.27; for WT and PS1/APP, respectively). As expected, BACE-1 was preferentially accumulated within dystrophies, surrounding Abeta plaques (Figure 4C). Furthermore, this alteration in BACE-1 level was also reflected by a higher BACE-1 enzymatic activity (Figure 4E). In parallel with these observations, the PS1-ctf fragment and PS1fl (practically undetectable in WT and at early ages in PS1/APP mice, Figure 4D) were also accumulated in aged PS1/APP mice (Figure 4D), in absence of variation in the transgene expression (data were normalized to expression levels detected in 6 months PS1/APP, n=6 for each age, 1.06±0.45, 1.0±0.69, 1.33±0.62 and 1.03±0.82 for 2, 6, 12 and 18 month PS1/APP, respectively). Although gamma-secretase is a multimeric protein and the increase in a single subunit could not directly reflect a parallel increment in the mature complex, it is of note that PS1-terminal fragments were exclusively generated when PS1fl was incorporated into the mature complex (see [34]). Thus, these data were consistent with an age-dependent increase in gamma-secretase. In fact, the gamma-secretase activity, determined as the rate of AICD production using fresh membranes isolated from different ages (in consequence, the substrate was the endogenous APP-CTF fragments, presented at each age) displayed a consistent increase in the aged PS1/APP samples (Figure 4F). It should be noted that, under these experimental conditions, we cannot discriminate if the increase in gamma-secretase activity was reflecting the accumulation of PS1-ctf or just an increase in substrate (C99 fragment, predominantly). Independently, the aged PS1/APP mice displayed an increased C99 processing capacity.

### Inhibition of lysosome increased APP-processing and Abeta accumulation in N2aAPPswe cells

We next evaluated, in vitro using APPSwedish transfected N2a cells [35], whether the APP metabolism was indeed affected by the inhibition of the intracellular proteolysis. For these experiments, the lysosomal or proteasomal/lysosomal activities were inhibited by the addition of Chloroquine (10 μM) or MG132 (5 μM). As expected, both lysosomal and/or proteasomal inhibition produced the accumulation of ubiquitinated proteins and the autophagosomal marker LC3-II (Figure 5A). Using the same protein extracts, we next tested whether the inhibition of proteasomes and/or lysosomes was reflected by alterations of the APP metabolism. As shown, both inhibitors produced a clear accumulation of APP-CTFs in APP N2a cells. In fact, C99 fragment and, more relevant, intracellular monomeric Abeta were accumulated after either treatment (Figure 5B). Furthermore, we also observed a parallel accumulation of BACE-1 and PS1-cft protein (Figure 5C). Therefore, these in vitro experiments demonstrated that the inhibition of lysosomal or proteasomal degradation processes produced the accumulation of APP fragments and Abeta production, in parallel with BACE-1 and PS1-ctf proteins. These data demonstrated that any misbalance in the correct intracellular proteolysis could induce the accumulation of APP products and, in consequence, increased the production of Abeta peptide.

**Figure 6:**
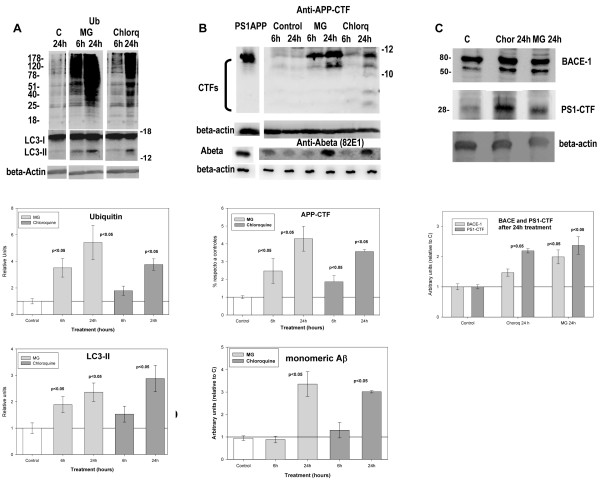
**Monomeric Abeta was preferentially accumulated in isolated synaptosomal fraction, from aged PS1/APP mice, whereas APP-C99 fragment preferentially localized in microsomes.A**) Microsomal and synaptosomal fractions were isolated from 6, 12 and 18 months WT and PS1/APP mice. Using these samples, we tested the distribution of LC3-II autophagosomal marker (a1), the APP-CTF fragments (a2) and the monomeric Abeta peptide (a3 and a4 for a longer exposure). ATP-synthase and b-actin were used as control of synapsomal integrity and protein loading. Quantitative analysis of western blots demonstrated the age-dependent accumulation of LC3-II (compared with the respective fraction in 6 month WT mice) (**B**) and C99 (as compared with 6-month-old PS1/APP microsomes) (**C**) in microsomal fractions. The monomeric Abeta was preferentially accumulated in the synaptosomal fractions (**D**). These experiments were repeated four times. **E**) Control experiments demonstrating the intrasynaptosomal origin of the monomeric Abeta. The tissue from a 12 month-old PS1/APP mice was divided, homogenized in absence (control) or in presence of 0.5% CHAPS + 0.5% DOC, and synaptosomes were isolated by Ficoll gradients. As shown, the disruption of cell membranes avoided the Abeta flotation on gradients (upper panels). On the other hand, tissue from 6 month-old WT mice were homogenized in absence (minus) of presence (plus) of synthetic Abeta42 and the synaptosomes were isolated. No Abeta were compartmentalized during homogenization.

### Age-dependent increase in Abeta accumulation in isolated presynaptic terminals (synaptosomes)

Finally, we also tested whether the observed age-dependent deficiencies in cytoskeletal proteins, lysosomal activity and APP-metabolism were reflected by modification of the APP fragment distribution in isolated synaptosomes. If the neuritic pathology in this model was predominantly, although not exclusive, of axonal origin, the observed modifications should produce the accumulation of APP-derived peptides (including Abeta) at the presynaptic terminals. In fact, we have recently reported the existence of presynaptic terminals containing multiple autophagic vesicles in PS1/APP mice [17]. To test this possibility, the homogenates were fractionated into microsomal and synaptosomal fractions. The microsomal fraction contained a heterogeneous population of vesicles of different origins whereas synaptosomes were principally enriched in presynaptic terminals.

We first compared the distribution of LC3-II protein, as a marker of autophagosomes, between microsomes and synaptosomes. As shown (Figure 6A, a1), in WT mice the LC3-II was abundant in microsomes whereas it was scarce in synaptosomes at any age tested (see also [17]). As expected, the LC3-II levels were higher in PS1/APP mice, as compared with WT, and increased with age in both fractions (Figures 6A and B). Thus, these data indicated the accumulation of autophagic vesicles at the presynapses [17] and also corroborated the existence of transport defects in PS1/APP mice.

We next analyzed the distribution of APP CTFs (C99 and C83) and Abeta between both fractions (Figure 6A, a2-4). Although the level of C99 increased clearly with age (in agreement with our previous data), the C99 content was patently higher in microsomes than in the synapsomal fraction. This preferential distribution was in agreement with the accumulation of BACE-1 in dystrophic neurites (Figure 4C) and the relative abundance of BACE-1 in microsomes (vs synaptosomes, data not shown). However, in spite of the large C99 accumulation, the monomeric Abeta peptide was scarce in microsomes and was preferentially accumulated at the synaptosomal fractions. It is noteworthy that, at early ages, synaptosomes accumulated low amount of monomeric Abeta (see Figures 6 a3, a4, D and E), whereas, at late ages, the monomeric Abeta peptide was highly concentrated at the synaptosomal fractions (63.6±7.5 fold as compared with 6 months, n=4; Figure 5D).

It could be argued that synaptosomal Abeta were of extracellular origin, due to contamination with plaques or by internalization during homogenization. To ascertain the intrasynaptosomal origin we have first tested whether the Abeta in the synaptosomal fractions was due to plaque contamination. As shown in Figure 6E (upper panels), the disruption of cellular membranes and the formation of synaptosomes, by homogenization of the tissue in presence of mild detergents (CHAPS plus deoxycholate), completely avoided the presence of Abeta on the synaptosomal fractions of the Ficoll-gradients. Second, the possible extracellular origin of Abeta, due to its compartmentalization on synaptosomes during homogenization, was tested by adding synthetic monomeric Abeta42 to WT samples in the homogenization buffer. As shown, Figure 6E lower panel, the synthetic Abeta was not recovered on the synaptosomal fractions of WT mice whereas it was clearly detectable on the soluble fractions. These control experiments demonstrated the intrasynaptosomal origin of Abeta.

## Discussion

In this work, we studied the progression of hippocampal neuronal pathology, in the PS1M146L/APP751SL model, that connects cytoskeleton and protein degradation dysfunction with dystrophy formation and synaptic Abeta production. As we reported previously, this model displayed the formation of axonal dystrophies, associated to extracellular Abeta deposition since early ages [17]. These dystrophies accumulated numerous vesicles of, among different origins, autophagic/lysosomal nature. Also, the axonal pathology was associated with the presence of aberrant presynaptic terminals in close proximity to Abeta plaques. In agreement with others [36-38] we observed that extracellular Abeta, probably by increasing the local calcium concentration, might produce cytoskeletal abnormalities that could induce transport defects (see also [39-43]). In fact, data presented in this work demonstrated the existence of a progressive increase in neurofilaments and tau hyperphosphorylation (SMI31, AT8 and AT100 epitopes). This increase was prevalently observed in dystrophies, surrounding the Abeta plaques. In conjunction with this apparent cytoskeletal dysfunction, we also observed a decrease in the levels of kinesin and dynein motor proteins. Taken together, these data strongly suggest the existence of a progressive, age-dependent, disorganization of the axonal cytoskeleton, which could impair the normal axonal transport, at local points in contact with extracellular Abeta deposits, (see also [25,43]). Furthermore, our data also demonstrated the accumulation of LC3-II and ubiquitinated proteins, principally at dystrophies. Whereas these observations could be interpreted as a simple accumulation of autophagosomes/lysosomes due to transport defects, our data also demonstrated the existence of a marked inhibition of the intracellular proteolytic activities. In fact, we observed a profound decrease in the cathepsin B and D activities and, in a minor extent, the proteasomal chymotrypsin activity. In parallel, we also observed a reduction in the mature forms of both cathepsins B and D in PS1/APP samples. The reasons that determined this marked and early proteolytic inhibition are unknown. It has been proposed that FAD mutations in PS1 impaired the maturation of V0a1 subunit of the vATP-ase [6]. However, as mentioned (see results), in our model, the V0a1 subunit displayed no apparent defects on its maturation. Furthermore, 6-month-old PS1M146L transgenic mice displayed no variations on cathepsin B activity. Thus, the observed decrease in cathepsin B and D activities seemed to be independent of the proton pump maturation (see also [44]). Nevertheless, our data were indeed compatible with defects on lysosomal acidification and/or maturation. In this sense, it has been also proposed that axonal transport was crucial for lysosomal maturation and function [33]. Thus, in our AD model, the cytoskeletal disorganization (probably mediated by extracellular Abeta and calcium misbalance) could also impair the lysosomal maturation (reflected by a decrease in the mature forms of both cathepsins tested) and, in consequence, the proteolytic function (decrease in the cathepsin activities). Further experiments should be done to ascertain this point.

We also analyzed the consequences of this neuritic pathology on APP processing and Abeta generation. In this sense, the overexpression of cathepsin B [45], the enhancement of cathepsin activities [9] or the positive lysosomal modulation [26] reduced the Abeta accumulation, the synaptic deficiencies and restored the cognitive function in transgenic mice. Thus, the progressive decrease of cathepsin activities and, in a minor extent, proteasomal activity, observed in our model, could be paralleled by an increase in the amyloidogenic APP processing. In fact, the observed age-dependent accumulation of hAPPfl and C99, together with the increase in BACE-1 and PS1-ctf proteins and their enzymatic activities, confirmed this proposition. Furthermore, our in vitro experiments also corroborated the rapid and progressive accumulation of APP derived C-terminal fragments, intracellular Abeta and both BACE-1 and PS1-ctf proteins after lysosomal and/or proteasomal inhibition (see also [46,47]). Therefore, based on these data, it is tempting to speculate that the formation of axonal dystrophies, probably due to the presence of extracellular Abeta [48], might produce a decrease in the intracellular proteolysis. This was paralleled by the accumulation of APP derived fragments inducing, in consequence, the production of higher amount of Abeta peptides (see also [49]). This self-progressing pathological scenario could be implicated in the progressive increase of Abeta and BACE-1 observed in AD patients (data not shown; see also [50]) and in the expansion of the pathology (see below).

Of note, our data also demonstrated the existence of a preferential accumulation of Abeta peptides in isolated synaptosomal fractions. Whereas this preferential accumulation could reflect the high gamma-secretase activity at the presynaptic terminals (data not shown but see [51]), these data also indicated that Abeta peptides were produced in compartment(s) different to, or in addition to, the autophagic vesicles [52]. This proposition was based on the observation that LC3-II, an autophagic marker, was preferentially concentrated in the microsomal fractions and it was scarce in synaptosomes. Although autophagic vesicles were indeed concentrated in pathological presynaptic terminals [17], the clear disproportion between LC3-II and Abeta in microsomes and synaptosomes, at all ages tested, strongly indicated a different compartmentalization. In this sense, it has been recently proposed that Abeta was synaptically produced and secreted from endosomal compartment [53]. Independently of the intracellular compartment, the synaptic production of Abeta peptides, observed in this work, could be implicated in the synaptic dysfunction in AD patients and in the pathological progression between synaptically connected areas. In this sense, it is widely accepted that the severity of the disease correlated better with the synaptic dysfunction rather than the plaque load. Thus, the age-dependent accumulation and, probably, release of Abeta peptides by presynapses could be directly implicated in the dendritic spine alterations observed in AD patients and in AD models. Furthermore, recent publications have been highlight the progression of Abeta [54,55] and tau [56,57] pathology between synaptically connected regions. In this sense, we have also reported the existence of a preferential Abeta deposition, microglial activation and neurodegeneration of principal neurons in layers V-VI of the entorhinal cortex [58]. However, these particular pyramidal cells did not express the transgenic hAPP and, in consequence, did not produce Abeta peptides. Thus, the presynaptic accumulation of Abeta peptides, reported here, might be implicated in the Abeta deposition and pathological spreading from Abeta producing region/layer into other brain regions.

In sum, our data demonstrated the existence of a progressive, age-dependent, cytoskeletal pathology (probably due to the extracellular Abeta deposition) that could be implicated in a reduction of the intracellular proteolytical processes. This impairment was associated to a progressive accumulation of APP derived fragments (and Abeta peptides) according with the increase of BACE-1 and gamma-secretase activities. This retard in the APP metabolism seemed to be directly implicated in the synaptic Abeta accumulation and, in consequence, in the pathology progression between synaptically connected regions.

## Methods

### Transgenic mice

Generation and initial characterization of PS1M146L/APP751sl (PS1/APP) tg mice has been reported previously [10,58,59]. Heterozygous PS1/APP double tg mice (C57BL:6 background) were generated by crossing homozygous PS1 tg mice with heterozygous Thy1-APP751SL mice. Only male mice were used in this work. Age-matched non-transgenic male mice (WT) of the same genetic background (C57BL:6) were used as controls.

Mice were first anesthetized with sodium pentobarbital (60 mg/kg), the hippocampi were dissected and immediately frozen and stored at −80°C until use. For immunohistochemistry, anesthetized mice were perfused transcardially with a paraformaldehyde-based solution (see details below). All animal experiments were performed in accordance with the guidelines of the Committee of Animal Research of the University of Seville (Spain) and the European Union Regulations.

### RNA and total protein extraction

Total RNA from mice hippocampi or cultured cells was extracted using Tripure Isolation Reagent (Roche) as described previously [10,11,27,58,59]. After isolation, RNA integrity was assessed by agarose gel electrophoresis. The yield of total RNA was determined by measuring the absorbance (260:280 nm) of isopropanol-precipitated aliquots of the samples. The recovery of RNA was comparable in all studied groups (1.2-1.5 μg/mg of tissue).

The protein pellets, obtained using the Tripure Isolation Reagent and isopropanol-mediated precipitation, were resuspended in 4% SDS and 8M urea in 40 mM Tris–HCl, pH 7.4 and rotated overnight at room temperature to get complete protein solubilization.

### Reverse transcription and real-time RT-PCR

Retrotranscription (RT) was performed using random hexamers, 4 μg of total RNA as template and High-Capacity cDNA Archive Kit (Applied Biosystems) following the manufacturer recommendations [10,27]. For real time RT-PCR, commercial Taqman™ probes (Applied Biosystems) were used for amplification. Alternatively, SYBRgreen dye and designed specific primers were used for amplification of human APP751 (forward: 5´-GGATATGAAGTTCATCATCA-3´; reverse: 5´-TCACTGTCGCTATGACAACA-3´), and human PS1 (forward: 5´-TGGCTCATCTTGGCTGTG-3´; reverse: 5´-ACCAGCATACGAAGTGG-3´). PCR reactions were carried out using either ABI Prism 7000 or 7900HT sequence detector systems (Applied Biosystems). A standard curve was first constructed for every assay, using increasing amounts of cDNA. In all cases, the slope of the curves indicated optimal PCR conditions (slope 3.2-3.4). The cDNA levels of the different mice were determined using GAPDH as housekeeper. Therefore, GAPDH amplification was done in parallel with the gene to be analyzed, and this dada used to normalize target gene results.

Independent of the analyzed gene, results were always expressed using the comparative Ct method, following the Bulletin number 2 from Applied Biosystems. As a control condition, we selected 6-month-old WT mice. In consequence, the expression of all tested genes, for all ages and mouse strains, was referenced to the expression levels observed in 6-month-old WT mice.

### Western blot

Western blots were performed as described [60]. Briefly, 5–20 μg of proteins from the different samples were loaded on 16%-SDS-tris-tricine-PAGE or 12%-SDS-tris-glycine-PAGE and transferred to nitrocellulose (Hybond-C Extra; Amersham).

After blocking, using 5% non-fat milk, membranes were incubated overnight, at 4°C, with the appropriate antibody: phospho-neurofilament (clone SMI-31; 1:1,000; Abcam), total-neurofilament (clone SMI-32; 1:1000; Abcam), phospho-Ser199:202-Thr205-PHF-tau (clone AT8; 1:1000; Pierce), phospho-Thr212-Ser214-PHF-tau (clone AT100; 1:1,000; Innogenetics), kinesin-1 heavy chain (1:1,000; Abcam), dynein-1 intermediate chain (1:1000; Millipore), ATP-synthetase-Beta (1:1000; BD Transduction Laboratories), Abeta peptide (clone 6E10; 1:5000; Signet), APP C-terminal (1:6000; Calbiochem), PS1 C-terminal (1:2000; Millipore), BACE-1 (1:1000; Abcam), V0a1-proton-pump subunit (1:1000; Synaptic Systems), LC3B (1:1000; Cell Signaling), cathepsin B (1:1000, Santa Cruz); cathepsin-D (1:5000; ABFrontier Co. Ltd), Lamp-1 (1:1000; Developmental Studies Hybridoma Bank; University of Iowa), ubiquitin (1:1000; Sigma-Aldrich) and Beta-actin (1:5000; Sigma-Aldrich). Membranes were then incubated with the corresponding horseradish-peroxidase-conjugated secondary antibody (Dako, Denmark) at a dilution of 1:8000. Each blot was developed using the ECL-plus detection method (Amersham).

For quantification, the scanned (Epson 3200) images were analyzed using PCBAS program. For normalization purposes, proteins were first estimated by Lowry and protein loading corrected by beta-actin. The intensity of bands from 6-month-old WT or PS1/APP (Figures 4A, E and F; Figures 5C and D) mice were averaged and considered as 1 relative unit. All other data were then normalized by the specific signal observed in 6-month-old WT or PS1/APP group or negative control for “in vitro” experiments.

### Enzymatic activity determination

BACE-1, Cathepsin B and D activities were determined using commercial kits (R&D Systems, Germany; Calbiochem, Germany and Sigma-Aldrich, respectively) following the manufacturer instructions. Briefly, fresh hippocampal samples were homogenized in the buffer supplied by the manufacturer or in PBS (for proteasome activity), centrifuged at 10,000xg (15 min at 4°C) and the supernatant (100–200 μg of protein per assay) was used. BACE-1 and cathepsin B and D activities were determined using substrates provided by the manufacturer

Proteasome chymotrypsin-like activity was determined as described [61]. Briefly, soluble fractions (30–50 μg of protein per assay) were diluted in 50 μM reaction substrate Suc-Leu-Leu-Val-Tyr-aminomethylcoumarin (AMC) (Sigma-aldrich), 0.1mM EDTA, 5mM DTT, 0.01% (w:v) CHAPS; 100mM NaCl; 1% (v:v) Glycerol, 50mM HEPES-KOH pH 7.5. Duplicated reactions were placed in a 96-well black polystyrene microplate (BD Transduction Labs.) and incubated at 37°C. Fluorescence was determined at excitation 360-380nm and emission 460-480 nm.

For each enzymatic assay, the fluorescence intensity was determined every 15 minutes (starting by the addition of the substrate) for a 1–2 hours final incubation time, using a Synergy HT Multi-mode microplate reader (Biotek). The activities were calculated from the maximal slope of the fluorescence intensity vs time curves and corrected by the amount of protein added. The results were then normalized by the activity observed in 6 months WT mice or 6 months PS1/APP mice (for BACE-1).

Also for each enzymatic activity, a reaction without substrate, a reaction without sample, and reaction with an inhibitor were used as negative controls. The inhibitors used were: Cathepsin B, Inhibitor Ref. 219385 (Callbiochem); Cathepsin D, pepstatin A Solution Ref. P3749 (Sigma-Aldrich); Proteasome 10 μM MG-132 (Sigma-Aldrich). Independently of the enzymatic activity assay, each experiment was repeated, at least, three times for each age and genotype.

Gamma-secretase activity was determined following previously described protocols with some modifications [62]. Briefly, membrane pellets from PS1/APP animals (n=6 per age) were thawed and resuspended (at 3 mg of protein per ml) in 150 mM Citrate Buffer, pH 6.4, containing protease inhibitors (Roche). Aliquots (150 μg of proteins) were used for each assay. As negative control, 100 μM L-685-458 gamma-secretase inhibitor (Calbiochem) was added prior assay. Samples were then incubated, at 37°C with orbital shaking at 400 rpm, for 2 hours. After incubation, membranes were sonicated (at 80 W for 30 seconds) and centrifuged at 30,000xg (30 minutes, 4°C). Supernatants were used to determine AICD production by western blot using anti-APP C-terminal as primary antibody.

### Tissue preparation for immunohistochemistry

After anesthesia with sodium pentobarbital (60 mg/kg), 6, 12, and 18-month-old control (WT) and PS1/APP tg male mice (n=4/age/genotype) were perfused transcardially with 0.1 M phosphate-buffered saline (PBS), pH 7.4 followed by 4% paraformaldehyde, 75 mM lysine, 10 mM sodium metaperiodate in 0.1 M phosphate buffer (PB), pH 7.4. Brains were then removed, post-fixed overnight in the same fixative at 4°C, cryoprotected in 30% sucrose, sectioned at 40 μm thickness in the coronal plane on a freezing microtome and serially collected in wells containing cold PBS and 0.02% sodium azide. All animal experiments were approved by the Committee of Animal Use for Research of the Malaga University (Spain) and the European Union Regulations.

### Immunohistochemistry

Coronal free-floating brain sections (40 μm thick) from 6 and 12–18 month-old control (WT) and PS1/APP mice were processed simultaneously in the same solutions and conditions to prevent processing variables. Sections were first treated with 3% H_2_O_2_/3% methanol in PBS and with avidin-biotin Blocking Kit (Vector Labs, Burlingame, CA, USA), and then incubated overnight at room temperature with one of the following antibodies: anti-APP-C-terminal rabbit polyclonal (1:20,000; Sigma-Aldrich), anti-phospho-Ser199:202:Thr205-PHF-tau mouse monoclonal (clone AT8; 1:500; Pierce) anti-ubiquitin rabbit polyclonal (1:2,000, Dako), anti-LC3 goat polyclonal (1:1,000, Santa Cruz Biotechnology), anti-BACE-1 rabbit polyclonal (1:1,000, Abcam) or anti-Lamp2 rabbit polyclonal (1:500, Abcam).

The tissue-bound primary antibody was detected by incubating with the corresponding biotinylated secondary antibody (1:500 dilution, Vector Laboratories), and then followed by streptavidin-conjugated peroxidase (Sigma Aldrich) diluted 1:2000. The reaction was visualized with 0.05% 3-3’-diaminobenzidine tetrahydrochloride (DAB, Sigma Aldrich), 0.03% nickel ammonium sulphate and 0.01% hydrogen peroxide in PBS. When required, immunolabeled sections were then incubated for 3 minutes in a solution of 20% Congo red. Sections were then mounted on gelatin-coated slides, air dried, dehydrated in graded ethanols, cleared in xylene and coverslipped with DPX (BDH) mounting medium. Specificity of the immune reactions was controlled by omitting the primary antiserum.

### Quantitative analysis of Abeta plaques-associated dystrophic neurites

The number of APP-immunopositive dystrophic neurites per plaque was quantified over Congo red stained Abeta deposits in sections from PS1/APP animals at young (4 and 6 month-old group) and old (12 and 18 months-old group) ages (n= 6/group; 5 sections per animal through the antero-posterior extent of the hippocampus). Quantification was done in CA1 subfield which was defined using a 10x objective and the number of dystrophic neurites was counted using a 100x objective in an Olympus BX61 microscope equiped with NewCAST software package (Olympus, Glostrup, Denmark). All plaques present in the CA1 region of each section were quantified. The number of dystrophic neurites per plaque was normalized to the mean plaque area to allow comparisons between groups.

### Synaptosome and microsome fractions isolation

Synaptosomal fractions were obtained basically as described previously [17,63]) with some modifications. Briefly, one mouse hemicortex was gently homogenized with a glass Dounce homogenizer in cold Buffer A (0.32 M sucrose, 1 mM EDTA, 1mM EGTA in 20 mM Tris–HCl pH 7.5, plus 1mM sodium orthovanadate, 50 mM sodium floride and a complete protease inhibitor cocktail (Roche) at a ratio of 40-50 mg of tissue per ml of buffer. This homogenate was first centrifuged at 1500 g and the post-nuclear supernatant was again centrifuged at 12,600 g for 20 minutes at 4°C to get the crude synaptosomes fraction. This pellet was resuspended in 13% (w:v) (final concentration) Ficoll PM400 (in buffer A) and layered on the bottom of a discontinuous gradient, composed by buffer A and 7% Ficoll (in buffer A). The gradient was centrifuged at 100,000g (45 minutes, 4°C) in a TLS-55 swimming bucket rotor (Beckman-Coulter), and synaptosomes were isolated at the 7.5–13% interface. After washing (twice with buffer A), the pellet of synaptosomes was resuspended in Buffer A. The possible contamination with vacuolated postsynapses was evaluated by testing the presence of tau (presynaptic) and MAP2 (postsynaptic) proteins. Results (not shown) indicated the existence of a minimal contamination with vacuolated postsynapses.

On the other hand, the microsomal fraction was obtained after additional centrifugation of the 12,600 g supernatant at 100,000 g (1 hour, 4°C) in a TLA-110 rotor (Beckman-Coulter). The pellet of microsomes was also resuspended in Buffer A. The protein content of the both synaptosomal and microsomal fractions was determined by Lowry.

### APPswe-expressing N2a cultures

APPswe-stably transfected Neuroblastoma cells were generously donated by Dr. Gopal Thinakaran (University of Chicago) [35]. N2aAPPswe cells were cultured in high glucose DMEM-Optimem (50%-50%) supplemented with 2 mM glutamine and 5% fetal bovine serum (PPA Company), in presence of Penicillin and Streptomycin (100 units/ml and 0.01 mg/ml respectively) and G418 (PPA Company) as clonal selection antibiotic (0.2 μg/ml) [35]. For cell drug treatments, a 0.2μm filtered stock solution of Chloroquine diphosphate salt (Sigma-Aldrich) or MG132 (Sigma-Aldrich) were diluted in the same media at a final concentration of 10 or 5 μM, respectively. This media was kept for 6 or 24 hours before collecting the cells and isolating RNA and protein as described above.

### Statistical analysis

Data were expressed as mean ± SD. The comparison between two mice groups (WT and PS1/APP mice) was done by *t* test. For comparison between several age groups, we used one-way ANOVA followed by Tukey post hoc multiple comparisons test (Statgraphics plus 3.1). As stated above, for most experiments, the different xgroups were compared with 6-month-old WT mice. In some cases (Figures 4A, E and F; Figure 5C and D) 6-month-old PS1/APP mice were used as reference. The significance was set at 95% of confidence.

## Abbreviations

AD: Alzheimer disease; APP: Amyloid Precursor Protein; Aβ: Amyloid β-protein; BACE-1: Beta-Secretase-1; CTF: Carboxyl-Terminal-Fragment; LC3: Microtubule-Associated Protein Light Chain 3; PS1: Presenilin-1.

## Competing interest

The authors declare that they have no competing interests.

## Authors’ contributions

MT, SJ, IC, VN and MV carried out the molecular experiments; R S-V, L T-E, and E M-S carried out the immunohistochemical experiments, JCD and MV participated in the design of experiment and revising the manuscript, AG and JV design the experiments, analyzed the data and wrote the manuscript. All authors read and approved the final manuscript.
